# Early Identification of Common-Source Foodborne Virus Outbreaks in Europe

**DOI:** 10.3201/eid0909.020766

**Published:** 2003-09

**Authors:** Marion Koopmans, Harry Vennema, Herre Heersma, Elisabeth van Strien, Yvonne van Duynhoven, David Brown, Marc Reacher, Ben Lopman, for the European Consortium on Foodborne Viruses

**Affiliations:** * National Institute of Public Health and the Environment, Bilthoven, the Netherlands; † Public Health Laboratory Service, London, United Kingdom

**Keywords:** outbreaks, foodborne virus, calicivirus, molecular epidemiology, gastroenteritis

## Abstract

The importance of foodborne viral infections is increasingly recognized. Food handlers can transmit infection during preparation or serving; fruit and vegetables may be contaminated by fecally contaminated water used for growing or washing. And modern practices of the food industry mean that a contaminated food item is not limited to national distribution. International outbreaks do occur, but little data are available about the incidence of such events and the food items associated with the highest risks. We developed a combined research and surveillance program for enteric viruses involving 12 laboratories in 9 European countries. This project aims to gain insight into the epidemiology of enteric viruses in Europe and the role of food in transmission by harmonizing (i.e., assessing the comparability of data through studies of molecular detection techniques) and enhancing epidemiologic surveillance. We describe the setup and preliminary results of our system, which uses a Web-accessible central database to track viruses and provides the foundation for an early warning system of foodborne and other common-source outbreaks.

Food-related illness is common worldwide, and bacterial pathogens have historically been associated with this mode of transmission. In recent years, however, the cause of most outbreaks of foodborne illness remained unknown, although a significant proportion were presumed to be viral (*1*). Additional research established the importance of viruses, especially the human caliciviruses belonging to the genus *Norovirus* (NV) (*2*). Transmission of these viruses is primarily from person to person, but numerous examples illustrate that NV are efficiently transmitted in food, water, or contaminated environmental surfaces. NV similar to, but not identical with, human strains have been found in cattle and in pigs (*3**,**4*). Studies in which viruses were molecularly characterized have shown that numerous variants co-circulate in the community but that occasionally shifts occur in which a single variant dominates over a wide geographic region (*5*). In 1995 to 1996, a worldwide epidemic was observed (*6*). The mechanism of emergence of these variants is unclear, but one hypothesis is that they represent widespread common-source events.

While it is clear that enteric viral infections are common, far less established is how common the foodborne mode of transmission is and how important it is in the epidemiology of these viruses. The challenge lies not so much in detecting outbreaks related to foodborne contamination at the end of the chain (the food handler in the nursing home or restaurant), because those are likely to be detected by routine outbreak investigation, with or without molecular typing. Linking NV outbreaks to common-source introductions nationally or internationally may be more difficult because of the high secondary attack rate that results from rapid person-to-person transmission. Thus, an initial seeding event will rapidly be masked by the occurrence of new cases or outbreaks, suggesting that person-to-person transmission is the primary mode of spread. The likelihood of detecting such seeding events relies on effective surveillance, which combines epidemiologic assessment of the outbreak and molecular typing to discover and track potential links between outbreaks. Such molecular tracing, however, requires knowledge on diversity of “resident viruses” in the region under study to be able to recognize unusual increases. Therefore, we established a combined research and surveillance network for foodborne viruses that was granted by the European Commission. This project group combines complementary expertise from the fields of diagnostic virology, molecular virology, epidemiology, and food microbiology to study modes of transmission of NV across Europe. Mapping these pathways allows better founded estimates of the proportion of illness that may be attributed to foodborne transmission and identification of high-risk foods, processing methods, or import and transport routes, which subsequently can be a focus of prevention programs. The data are important for assessing the risks associated with consumption of certain food items. Essential to the success of this project is the establishment of a common, central database, which is now used by all partners to compare data across Europe as soon as they are available. We describe this project and results from its first 18 months of operation.

## Materials and Methods

The network is a collaboration among 12 laboratories in 9 countries in Europe to allow more rapid and internationally harmonized assessment of the spread of foodborne viral pathogens. The project is coordinated by the National Institute of Public Health and the Environment in Bilthoven, the Netherlands. Participants are virologists and epidemiologists with active research programs in (foodborne) enteric viruses from Spain (Barcelona, Valencia, Madrid), Italy (Rome), France (Nantes, Dijon), Germany (Berlin), the Netherlands (Bilthoven), the United Kingdom (London), Denmark (Copenhagen), Sweden (Solna), and Finland (Helsinki). In addition, groups from Slovenia and Hungary participate.

The overall objectives for the complete study are as follows: 1) to develop novel, standardized, rapid methods for detection and typing of enteric viruses, particularly NV, to be used in all participating laboratories; 2) to establish the framework for a rapid, prepublication exchange of epidemiologic, virologic, and molecular diagnostic data; 3) to study the importance of enteric viruses as causes of illness across Europe, with a special focus on multinational outbreaks of infection with NV and hepatitis A virus; 4) to provide better estimates for the proportion of NV infections that can be attributed to foodborne infection; 5) to determine high-risk foods and transmission routes of foodborne viral infections in the different countries and between countries; 6) to describe the pattern of diversity of NV within and between countries and identify potential pandemic strains at the onset; and 7) to investigate the mechanisms of emergence of these strains, including the possibility of spillover from animal reservoirs

The central research goal is to better understand the mechanisms of emergence of variant NV strains. We hypothesized that the observed epidemic shifts might be caused by displacement of endemic variants attributable to a large seeding event with a variant that subsequently spread through the population by secondary and tertiary waves of transmission, or possibly by a smaller seeding event of a highly transmissible new variant, generated by genetic mutation or recombination. To address these questions, we built a European surveillance structure for outbreaks of viral gastroenteritis, including food- or waterborne outbreaks. The first phase of the project, described in this report, was designed to review existing surveillance systems for viral gastroenteritis, to design and agree on a minimum dataset for collection during the second phase of the project; to review and evaluate currently used methods for detection and genotyping of NV with the aim of standardizing methods for virus detection in gastroenteritis outbreaks; and to build a database of combined epidemiologic and virologic data for use by all participants. The overriding aim was to facilitate the early detection of potentially emerging variant strains. Upon completion of this phase, we will begin “ enhanced surveillance”, i.e., standardized surveillance for viral gastroenteritis outbreaks to study objectives 4–7.

## Results

### Review of Current Methods in Europe

From the outset, it was recognized that the best approach in developing an international surveillance scheme for foodborne viruses would not be the standardization of practice, but rather the harmonization of existing practices. To achieve this, a number of surveys were undertaken to determine diagnostic capabilities, genotyping techniques, and the status of surveillance of viral gastroenteritis outbreaks among project participants. The results of these surveys are highlighted below.

### Virus Detection and Genotyping

The scale of diagnostic capability in laboratories varies widely, and a range of diagnostic tests (electron microscopy, reverse transcription–polymerase chain reaction [RT-PCR], and enzyme-linked immunosorbent assay) and characterization methods are used (including heteroduplex mobility assay, reverse line blot, microplate hybridization, and sequencing (*7*–*9*). Laboratories in all countries now use molecular techniques (RT-PCR) for NV detection (*10*).

A comparative evaluation of RT-PCR assays was done by analysis of a coded panel of stool samples that had tested positive (81 samples) or negative (9 samples) for NV. Samples provided by four laboratories were included, as well as a samples representing the currently known diversity of NV genotypes. Full details of this study have been published (*11*). This evaluation showed that no single assay is best, although sensitivities range from 55% to 100%. Most differences were seen when comparing assay sensitivities by genogroup. Based on pre-set scoring criteria (sensitivity, specificity, assay format, length of sequence), one primer combination was ranked as the assay of first choice for laboratories starting surveillance, and protocols and reagents have been made available to all participants on request.

On the basis of the aggregated data from the sequence database, alignments were made of the regions in the viral RNA that contain the primer-binding sites for the set of primers with the highest ranking for the diagnostic evaluation to generate more optimal designs of primers (*12*). These primers, protocols, and reference reagents have been made available to several groups in the field.

### Outbreak Investigations

While all countries in the network now have the diagnostic capability to recognize outbreaks of NV, the structure of their national surveillance differs and therefore, so do the epidemiologic data collected on viral gastroenteritis (*10*,*13*). Some countries investigate outbreaks of gastroenteritis irrespective of the size or possible mode of transmission (United Kingdom, the Netherlands); others primarily investigate outbreaks that appear as foodborne from the onset (Denmark, France) (*10*). Similarly, coverage of the laboratories involved ranges from regional (Italy) to national, although different levels of underreporting are likely to exist (*10*) These differences, as well as differences in the laboratory test protocols, will be taken into consideration when interpreting aggregated data in the later stages of the project. For the purposes of comparing data across Europe, however, the key finding was that most countries maintain a national database of NV outbreaks (as opposed to individual cases). Although the proportion of the population that these databases effectively survey and the completeness of clinical information collected vary, we recognized that we could network national outbreak surveillance by agreeing on a minimum dataset. That dataset would include the causative organism, mode and place of transmission, diagnostic results, case details, food vehicles, and viral typing information.

Also agreed upon were clinical definitions of a case and an outbreak of viral gastroenteritis based on Kaplan’s criteria (*14*), as follows. A case of gastroenteritis was defined as a person seen with 1) vomiting (two or more episodes of vomiting in a 12-hour period lasting >12 hours), or 2) diarrhea (two or more loose stools in a 12-hour period lasting >12 hours, or 3) vomiting as defined in 1) and diarrhea as defined in 2). An outbreak was defined as follows: 1) Patients living in more than one private residence or resident or working in an institution at the time of exposure; 2) cases linked by time and place; 3) vomiting in >50% of total cases (*14*); 4) mean or median duration of illness of total cases from 12 to 60 hours; 5) incubation period (if available) of total cases between 15 and 77 hours, usually 24–48 hours (*14*,*15*); and 6) testing of stool specimens for bacterial pathogens. (This step is not mandatory;, however, if tested, all specimens should be negative for bacterial pathogens.)

### Development of Database

A major goal of the first year was to build a database into which historic information present in the participating institutes would be collected. The rationale behind this was that by combining this existing information, new observations (on seasonality of outbreaks or patterns of emergence of new variants, for example) might be possible. Without harmonization of data collection, the comparative analysis would clearly be limited. The historic database, however, also served as a pilot phase because the definitive format of the database is used in the enhanced surveillance program. Participants who had historic collections of sequences were asked to submit these, along with additional epidemiologic information, as described in Table 1. Data were entered by using the Bionumerics (BN) package (Applied Maths, Ghent, Belgium), which allows storage, comparative analysis, and clustering of combined epidemiologic and biological experimental data (e.g., sequences, reverse line blot results, enzyme immunoassay data). The entries were either uploaded from the public domain or submitted as unpublished sequences from participating laboratories. Publicly available sequences were included to provide a customized report for database searches, e.g., genotype to which the sequence belongs.

**Table 1:** Requested data fields for entries of viral sequences in the historic database^a^

Field name	Field type	Mandatory field
Country	A	Yes
Institute	A	Yes
Reference no.	A	Yes
Type of virus (NV, SV, HAV, HEV, rotavirus group C?)	N	Yes
Sequence identifier (e.g., GenBank accession no.)	A	No
Method no.	N	No
Genotype/subtype	N	No
Sequence pol region /VP1/2a	A	Yes or no?
Sequence capsid region	A	
Source of isolate (human, calf, swine, environmental, other)	N	Yes
Specimen	N	No
Epidemiology	N	No
Vehicle (food, water, person-to-person, zoonotic, not known)	N	No
National region	A	No
Date of receipt in reference laboratory	D	Yes
Age	N	No
Sex	N	No
Travel associated	N	No
Travel destination	A	No

^a^NV, Norovirus; SV, Sapovirus; ASV, astrovirus; HAV, hepatitis A virus; HEV, hepatitis E virus; A, letters and numbers; B, numbers; D, data.

Since September 2001, participants have been able to access the database directly through a password-controlled Internet connection. At present, the database contains >2,500 entries, mostly on NV, but including some hepatitis A virus, astrovirus, and Sapovirus (Tables 2 and 3). Upcoming variants will first be subjected to a search of the historic database to determine if the viruses have been seen before in Europe. An automated search tool is available and has been made accessible through the Internet to participants. Partners interested in analyzing the data can obtain the complete dataset, provided they adhere to the confidentiality agreements signed by every partner. Interested parties outside the project group can access the database under certain conditions through the coordinator or one of the participants. This access is not restricted to groups in the participating countries. The limiting factor is the target region used for virus characterization, which has not been standardized globally. A database search will be performed upon request (for groups outside the network). Results are then communicated to them and to the person who submitted any matching sequences. After that initial linking, follow-up discussions and investigations of possible common-source events can be done by the groups involved.

**Table 2:** Number of entries in the database by year of detection for selected viruses, as determined by reverse line blot typing^a^

	N	HAV	SLV	ASV	NLV	NLV capsid	NLV pol	NLV cap and pol	RLB	animal
Not dated	364	69	4	7	284	152	119	9	1	2
1989 and before	102	68	2	-	32	15	23	7		
										
1990	43	31	1	-	11	7	10	6		
1991	5	-	-	-	5	2	5	2		
1992	11	-	2	-	9	3	7	3		
1993	21	-	2	-	19	14	26	11		
1994	45	-	1	-	44	20	38	14		
1995	49	-	-	2	47	15	41	9		
1996	122	-	1	4	117	18	100	2		
1997	122	-	-	1	121	8	117	5	3	6
1998	158	-	2	6	152	19	130	11	6	13
1999	531	44	2	26	485	39	366	32	129	7
2000	446	40	2	-	404	61	380	41	26	6
2001	419	5	2	2	410	52	396	52	-	-
2002	155	-	-	-	155	3	155	3	-	

^a^HAV, hepatitis A virus; SLV, Sapporo-like virus; ASV, astrovirus; NLV, Norwalk-like viruses.

**Table 3:** Number of entries in the database by country of submission^a^

Country	No. of entries	HAV	ASV	NLV	SLV
Argentina	9			9	
Austria	17			17	
Canada	20			20	
Switzerland	23			23	
Czech Republic	1	1			
Germany	167	1		166	
Spain	33	18		15	
Finland	90			90	
France	216		24	185	7
Great Britain	509	1		505	3
Ghana	2			2	
Hong Kong	7			7	
Japan	363			360	3
Korea	1			1	
Mexico	1			1	
Netherlands	743	87	24	632	
Norway	13			13	
New Zealand	5			5	
Russia	8			8	
South Africa	9			5	4
Sweden	101			100	1
Turkey	1	1			
United States	67	6		59	2

^a^HAV, hepatitis A virus; ASV, astrovirus; NLV, Norwalk-like viruses; SLV, Sapporo-like virus.

### Prospective Enhanced Surveillance

#### Comparative Evaluation of Diagnostic/Genotyping Methods

The different PCR primers used among the European group all target a highly conserved region within the viral polymerase gene. Sequences of the amplicons from the various diagnostic PCRs overlap and therefore, can be compared to gain inferences on the molecular epidemiology and the spread of NV variants (*11*). Rapid characterization techniques, notably the reverse line blot (*9*) and heteroduplex mobility assay (*7*), are also used within the network; the typing data generated by these techniques can also be accommodated by the database.

### Comparative Evaluation of Data

After agreement on a minimum epidemiologic and virologic dataset, we made a standard Web-based questionnaire available to all participants behind a password-protected site (available from: URL: www.eufoodborneviruses.net). Using Web-based Active Server Pages (ASP) technology, investigators have full access to the secure outbreak database (Figure). Investigators are asked to enter information that is available as soon as an investigation begins on an event occurs that meets the outbreak definition. A unique reference number is assigned to each outbreak, which is the key used to access records and to update diagnostic or typing data, for example, as an investigation continues.

**Figure:**
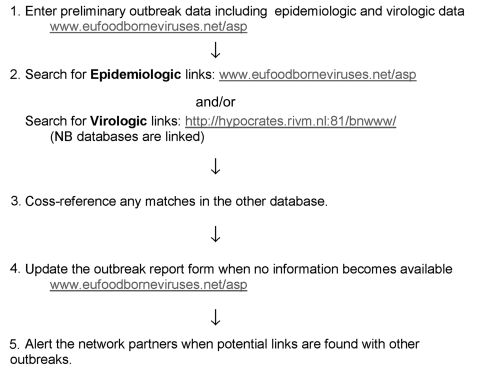
Timeline of Web-based reporting of epidemiologic and virologic data and interrogation of the database for the Foodborne Viruses in Europe Group.

The database also collects information on the level of evidence (i.e., microbiologic, epidemiologic, circumstantial) implicating food or water as a mode of transmission. Pop-up windows are used to define these criteria, since a range of public health scientists use the system. Other features of the ASP technology, including drop-down menus, are used to standardize the data collected. Descriptive information from outbreaks (number of people exposed, number of people ill, number of controls infected, symptoms) is collected when possible, to allow comparisons of the clinical characteristics of different NV genotypes. Preliminary data suggest that such differences exist.

One of our main scientific objectives is to explain the mechanism behind the emergence of new variant strains. Essential for the early detection of such emerging variants is a rapid reporting network. The initial suspicion of “something strange” may be from clinicians who investigate outbreaks (e.g., a sudden increase in the number of reports), or from one of the laboratories (e.g., finding the same variant in several outbreaks). The central database is used to facilitate both types of reports. The real power in this format of data exchange is that immediately after entry or update of information, the data are in the database and can be accessed by other collaborators. The database can be searched for common virologic (sequence) or epidemiologic (e.g., a food vehicle) characteristics that would trigger further investigation of links between outbreaks.

### Recognition of International Outbreaks

This model has proved successful in recognizing a number of internationally linked events. Clusters of cases in Denmark, Finland, and the Netherlands were all linked to oysters imported from France. Another foodborne outbreak traced in part through the network followed the concluding dinner of an international conference in Finland. Symptoms began the day after the conference, when many attendees had returned to their home countries. Approximately 40 persons were affected, and the same NV variant (Melksham) was detected from cases in Finland, Sweden, and the Netherlands. A dessert item was implicated by cohort study. Importations of hepatitis A from Peru into Spain and from Ibiza, Spain, to Germany have also been recognized through the network. Full details of these outbreaks will be published elsewhere.

## Discussion

Microbial food safety is considered an important public heath issue but historically has focused on control of bacterial contamination. Several recent publications, however, show that outbreaks of foodborne infection attributable to viruses are common and may in fact be an important public health concern for several reasons: most clinical laboratories involved in outbreak investigations do not have access to routine diagnostic methods for detecting NV, user-friendly methods for use in these laboratories are only now becoming available and need to be validated, foodborne transmission of NV is quite common, and food microbial quality control largely relies on indicators for the presence of fecal bacteria, which may not correlate with the presence of enteric viruses (*2*,*16*). Although foodborne viruses are increasingly studied, no validated methods yet exist for reliably detecting them in food items. In all, these facts indicate that through foodborne transmission an enteric viral pathogen (NV) can escape detection, possibly resulting in large epidemics.

In the United States, molecular detection techniques are being implemented in state public health laboratories under the guidance of the Centers for Disease Control and Prevention (CDC), which is building an infrastructure for reporting of outbreaks of food-related illness attributable to enteric viruses (Calicinet). In Europe, no central institute yet exists with the authority to do this, so the best efforts to date are the voluntary disease-specific surveillance networks, such as Enternet (which monitors trends in foodborne bacterial pathogens), and the European Influenza Surveillance Scheme (designed to monitor influenza virus activity across Europe) (*17*,*18*). We have built such a surveillance network for enteric viruses, using NV as a target organism. NV was an obvious choice: an increasing number of publications illustrate that it is one of the most important causes of outbreaks of gastroenteritis, including food- and water-related outbreaks (reviewed in 2). CDC estimates that up to 66% of all food-related illness in the United States may be due to NV (*19*). From a community-based case-control study in the Netherlands, risk-factor analysis for NV, based on information collected throughout a 1-year cohort study, suggested an association between NV and a complex score that was used as a proxy for food-handling hygiene. On the basis of this approach, an estimated 12% to 15% of community-acquired illness may be due to food- or waterborne modes of transmission (with 85% attributed to contact with a symptomatic person in or outside the household) (de Wit et al., unpub. data). The proportion of foodborne outbreaks reported in the countries participating in our network ranges from 7% to 100%, but that range merely reflects the differences in the selections used in the different surveillance systems and cannot be used to estimate the true impact of foodborne illness caused by NV (*10*). While definitive data still need to be collected, the consensus is that NV is an important cause of food-related infection and disease.

Foodborne transmission of viral gastroenteritis has not historically been acknowledged as a public health priority, which means that our surveillance system is inevitably restricted to groups that already have an active program in the field. Ideally, we would like to build a network of national institutes represented by both epidemiologists and microbiologists involved in outbreaks of viral gastroenteritis; however, at present this ideal is not possible for all of Europe. By networking the existing information, assessing comparability of data through studies of primers and protocols used, and examining data from current surveillance, we are able to paint a bigger picture from the fragmented information that is available.

The standardized outbreak questionnaire, accessible through the Internet, is designed to collect a minimum dataset about all outbreaks. However, participants who perform more detailed epidemiologic or virologic investigations can also submit additional data. The minimum dataset will suffice to answer the basic questions for the surveillance, i.e., what is the reported incidence of NV outbreaks across Europe, and which proportion is considered to be due to food- or waterborne transmission.

A key feature of any disease surveillance system is its use as an early-warning tool, in this case for international common-source outbreaks. To facilitate this, several features were included in our database setup. Information on outbreaks can be updated with new information as it comes in, to avoid piling up information until the outbreak investigation has been completed (which may be months later). Both the epidemiologic data and laboratory data (mostly sequences) can be searched easily. Thus, participants can be alerted to similarities in disease profiles (e.g., outbreaks with imported fruits) or in sequences. Either signal can lead to contacts between participants to discuss possible indications for a joint investigation. Crucial in this discussion was the issue of confidentiality, both for patient and product information, and for data from investigations. The present modus operandi is that each participant signs a confidentiality agreement, which states that data submitted to the database are owned by the person submitting them (subject to each participant’s national regulations on patient and laboratory data); specific patient and product information is not entered into the database. If necessary for outbreak investigations, the groups involved will decide on a case-by-case basis what information may or may not be used by the consortium. Participants can obtain the complete information from the database for their own analysis, or choose to use it as a search tool and rely on the analysis done by a scientist employed on this aspect of the database, who is stationed in Bilthoven, the Netherlands. So far, five international outbreaks have been detected because of the network.

The food distribution chain in Europe is complex, and therefore the transmission of viruses across borders can occur by means of contaminated food. The surveillance network described here allows early detection of international common-source outbreaks of foodborne viruses. Most of the work to date has involved harmonization of methods for investigating outbreaks and detecting the viruses causing these outbreaks, as well as the development of a database system that facilitates the exchange of information between laboratories and institutes involved in viral gastroenteritis research and surveillance. The system’s strength is that it combines basic epidemiologic and laboratory data into a searchable repository. This network has demonstrated its potential to recognize transnational outbreaks. However, the network is inherently limited by the quality of data available at the national level, which is a reflection of the priority given to foodborne viruses. At present, we are undertaking a 2-year enhanced surveillance project to study the frequency and modes of transmission of viral gastroenteritis outbreaks across Europe.
